# Analysis of integrated UWB MIMO and CR antenna system using transmission line model with functional verification

**DOI:** 10.1038/s41598-022-17550-z

**Published:** 2022-08-19

**Authors:** P. Prabhu, Malarvizhi Subramani, Kyung-sup Kwak

**Affiliations:** 1 Department of ECE, SRM Institute of Science and Technology, Katankulatur, Chennai, Tamilnadu 603203 India; 2 UWB Wireless Communications Research Center, Inha University, Incheon, 402-751 South Korea

**Keywords:** Engineering, Electrical and electronic engineering

## Abstract

This paper discusses the equivalent circuit model and functional verification of an integrated antenna system as its main focus. The integrated antenna system consists of two independent antenna systems, namely the Cognitive Radio antenna and the Ultra Wide Band Multiple Iinput Multiple Output antenna. This article is split into two parts: The first part discusses the equivalent circuit of an integrated antenna system by optimizing the RLC values. The developed lumped equivalent circuit model produces the NB resonant frequencies, which is the same as the S-parameter obtained through EM simulation. The second part of this paper aims to discuss the experimental verification of an integrated CR antenna system with Bayesian learning-based spectrum sensing algorithms using Universal Software Radio Peripheral devices. Real-time sensing and communication functionalities are visualized in the LABVIEW monitor. The integrated antenna system is fabricated and measured after the simulation.

## Introduction

Recently, MIMO technology, Cognitive Radio (CR) technology, and integrated independent antenna systems attracted antenna researchers due to effective performance, compact size, low cost, and easy implementation. In 2002, the Federal Communications Commission (FCC) proposed many solutions to improve spectrum utilization efficiency. According to the usage of idle channels and spectrum white space, they are divided into three groups. They are namely spectrum reallocation, sharing, and leasing. Spectrum sharing is classified as open spectrum sharing, hierarchical spectrum sharing, and dynamic spectrum allocation. In addition, hierarchical spectrum sharing is accomplished in two ways. The first way is spectrum underlay, and the second way is spectrum overlay. In the spectrum overlay method, the Cognitive Radio (CR) technology is employed to exploit free channels or gaps in the spectrum. In a wireless environment, the licensed spectrum is a license fee paid by the main user, but some users have not always used the licensed spectrum. Hence, CR technology allows you to search for idle channels in the wireless environment regularly. If the cognitive system recognizes the free channel, the secondary user will use it for other wireless communication applications until the primary user needs it. Whenever the primary user tries to occupy a spectrum that was previously emptied, the secondary user changed to another working frequency or the communication will end until the next spectral hole is identified. The CR system utilizes the UWB antenna and Narrow Band (NB) antenna for sensing and communication purposes, respectively. Considering the structure of the antenna, the planar monopole antenna has the advantages of being easy to integrate with a flat structure, easy to manufacture, and small in appearance, so it has been widely used in CR technology. Sensing UWB antennas need to operate at frequencies of 3–10.6 GHz or higher than 10.6 GHz, but the FCC licenses frequencies up to 10.6 GHz, and the FCC does not license spectrum at frequencies after 10.6 GHz. The single band or multi-band NB communication antennas used for communication purposes. In recent years, re-configurable UWB/NB antennas have been used in CR technology. As mentioned above, the sensing antenna can sense multiple white spaces in the frequency spectrum atone time. Therefore, the re-configurable NB antenna needs to be continuously switched from one frequency to another frequency to communicate through identified multiple white spaces. Thus, the design of a multi-state switching re-configurable antenna is very complicated. Similarly, recently, research on MIMO antennas has also been rapidly developed. Multiple Input Multiple Output (MIMO) technology is resolving multipath fading and improves the system’s channel capacity using exiting spectrum^1,2^. Furthermore, UWB MIMO antenna^3,4^, Band notched UWB antenna^5–9^, Re-configurable Cognitive Radio (CR) antenna^10–15^, integrated CR antennas^16–19^, dual polarized metasurfraces^20,21^ and equivalent circuit analysis of UWB/NB antenna^22–26^ mentioned in the literature.  Reconfigurable antennas, on the other hand, have the following drawbacks: The biasing circuit is difficult to build, the toggling is influenced by in homogeneity, and the PIN diode needs extra power. The integrated antenna system^27^ provides solution for the above mentioned problems. There are two independent antennas in the proposed integrated antenna systems^27^. For the transmission line model analysis and functional verification we have considered our designed antenna^27^. First, part of the antenna system includes the CR antenna(UWB and NB) and second, part includes the UWB MIMO antenna^27^. The UWB antenna for detection and NB antennas to execute communications. The integrated CR and MIMO antennas enhance spectrum utilization and diversity performance because of their dual-polarization. For both sending and receiving, the UWB MIMO antenna radiators are constantly active, but sensing UWB radiators are always active^27^. In addition, the narrowband antennas are idle, but if the UWB antenna detects white space, the associated NB antenna is active for communication^27^. This article introduces the equivalent circuit model of integrated antenna system.

### Motivation, and contribution

As of now, many of the equivalent circuit models have been implemented for patch antenna, stacked patch antenna and notch filter. Only a few researchers have implemented equivalent circuit models for UWB antennas. In^28^ ,^29^ the transmission model is implemented for simple and stacked general patch antenna was carried out and equivalent circuit of the planar CR antenna system was carried out in^22^, but this article discuss the equivalent circuit model of the entire antenna system^27^. Also, the novel contribution of this work is this is the first article discussing the transmission line equivalent circuit model of entire integrated antenna system (Narrow band antenna, notch filter and UWB antenna) and transmission line equivalent circuit analysis done for the narrow band antennas, notch filter, and UWB antenna.

The main contributions of the proposed hardware implementation is as follows.

- First, we have designed an integrated CR and UWB MIMO antenna^27^.
- The UWB signal is transmitted by the transmitting antenna and received by the UWB sensing antenna (CR antenna), where the sending and receiving antennas are connected to two different USRP’s.
- From the received UWB spectrum, the white spaces are identified using Bayesian learning-based wideband spectrum sensing algorithms in MATLAB software.
- The proposed system hardware implementation is effective because it can be easily implemented with less hardware. Also, it has effective spectrum sensing and white space detection.
- Also, the MIMO antenna real time performance is analyzed using two element UWB MIMO antenna.

## Equivalent circuit model of integrated(Hybrid) antenna system

The UWB radiator with and without band notch, and narrow band antenna design is carried out in^27^. As a continuation of^27^ in this paper we have carried out detailed lumped circuit model analysis of each independent antenna system and real time implementation of the integrated antenna system.

### Equivalent circuit of narrow band(NB) antennas

**Figure 1:**
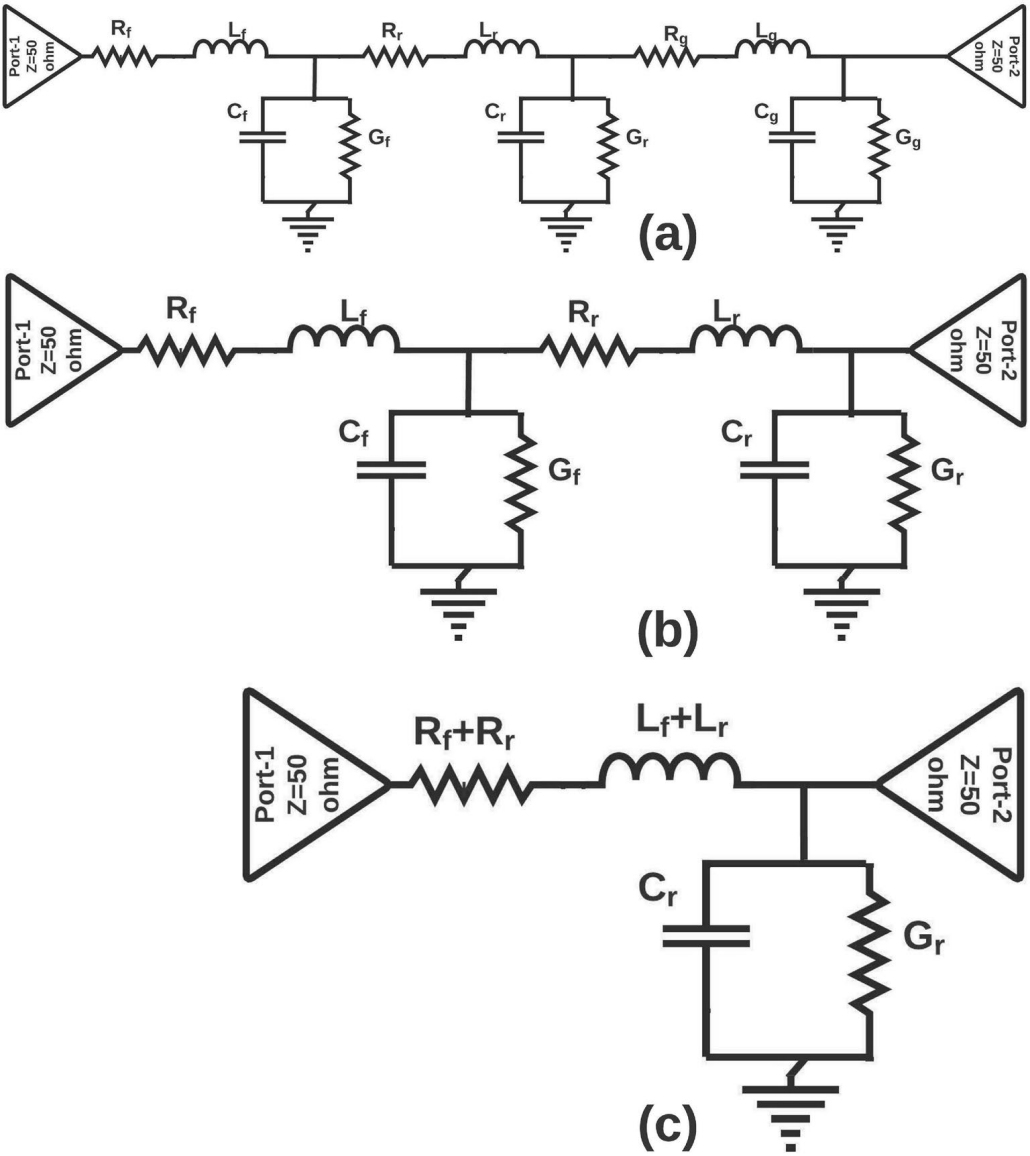
(**a**) Equivalent circuit model of general patch antenna (**b**) Mircrostrip antenna after Ist assumption, (**c**) Mircrostrip antenna after IInd assumption.

This section describes the lumped equivalent circuit model of NB antennas. In general, an antenna is a combination of linear and passive elements network. The antenna’s overall equivalent model can be obtained using lumped elements such as resistance (R), capacitance (C), inductance (L) and conductance (G). Any conducting element of the antenna is considered to be a combination of R and L in series as it provides resistance and inductance in a reason of conductivity. Besides, the conductor losses are denoted by R. It is assumed that the capacitor (C) and the conductance (G) are connected in parallel in the antenna because a dielectric material separates the upper and lower conductive layers. Further, when a conductive material two conductors, some capacitance occurs, and G is taken into account due to the dielectric loss. In addition, in order to consider fringe fields and plane waves, a small amount of inductance is taken into account. By considering this, inductance can improve the accuracy of the approximate lumped equivalent model. Initially lumped equivalent model of the fundamental patches (circular, rectangular, square, and other shapes) antenna is presented. However, it is only used for patches that have no slot. The same procedure is followed for the proposed NB antennas. In general, a patch antenna has three conducting elements such as feed line, radiating patch, and the ground plane. These three conducting layers are approximated by three series R and L combinations since each conducting layer form one series R and L combinations. Figure 1a shows the feed line, ground plane, and radiating patch equivalent circuit model represented by R$_{f}$, L$_{f}$, R$_{g}$, L$_{g}$, and R$_{r}$, L$_{r}$, respectively. In addition, the equivalent circuit model between the ground and feed is represented by the parallel combination of C$_{g}$ and G$_{g}$. Similarly, the parallel combination of C$_{r}$ and G$_{r}$ represents the equivalent circuit model between the ground and the radiating patch. The 50 $\Omega$ matched terminator is used to terminate the input port, while the radiating patch is closed with a 377$\Omega$ free space inherent impedance as shown in Fig. 1a. Equivalent model is simplified by using two assumptions, (i) The current flowing through the ground plane is very small compared to the current flowing through the radiating patch, so the series combination of R$_{g}$ and L$_{g\ }$of the ground plane is ignored. (ii) Shunt combination of capacitance C$_{g}$ and conductance G$_{g}$ formed between the feed line and ground plane, they are ignored because they contribute very little C and G. Due to this, the R(R$_{f}$+R$_{r}$) and L(L$_{f}$+L$_{r}$) of the feed line and patch is connected in series, and both are added. Eventually, the simplified equivalent circuit model is shown in Fig. 1b,c; it is obtained after considering two assumptions. Based on the second assumption, the resistance R$_{f}$, R$_{r\ }$and L$_{f}$, L$_{r}$ is represented by (R$_{f}$+R$_{r}$) and (L$_{f}$+L$_{r}$), as shown in the Fig. 1c.

**Figure 2:**
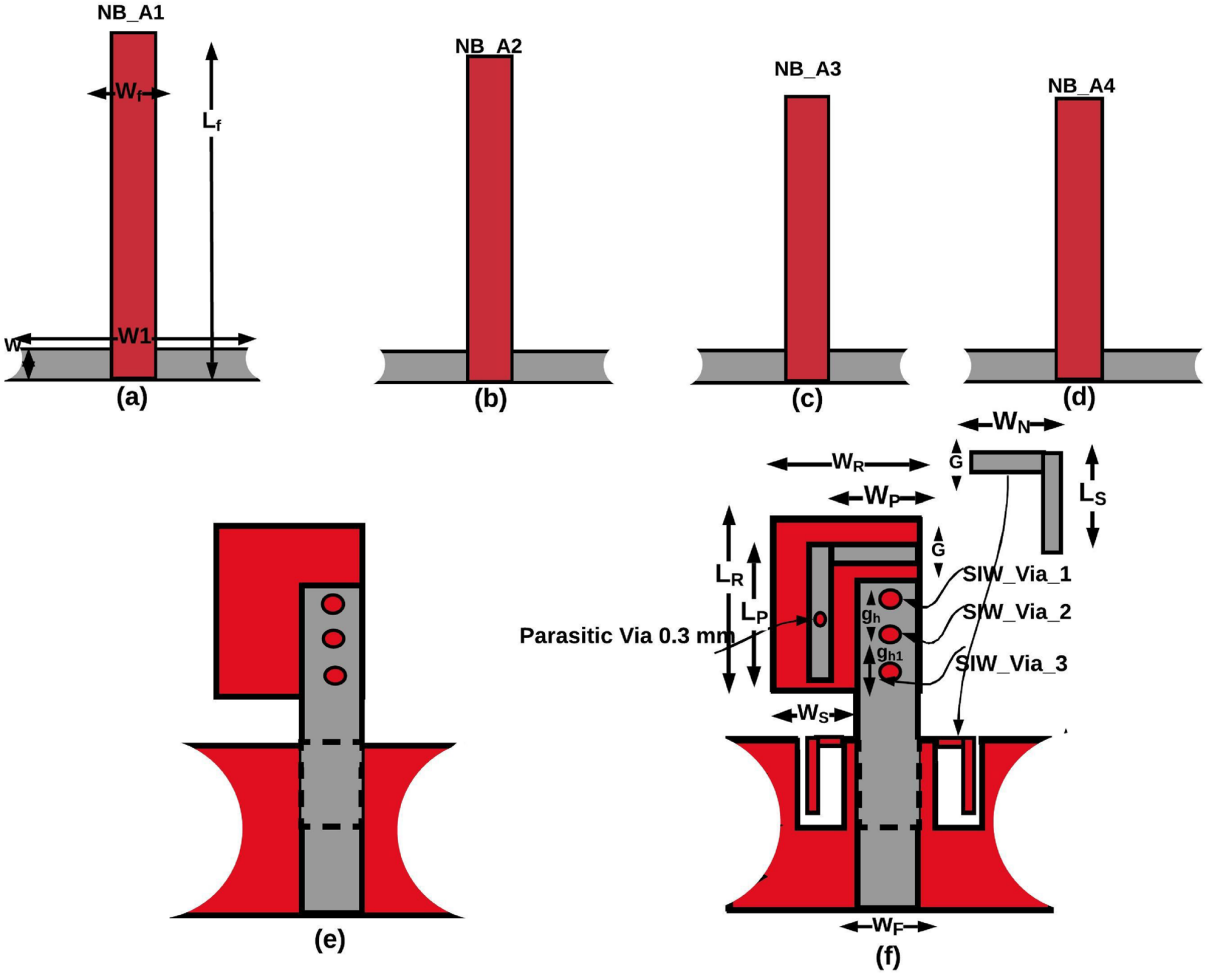
(**a**)–(**d**) Narrow band antennas, (**e**) Substrate Integrated Wave guide (SIW) based UWB antenna, (**f**) Substrate Integrated Waveguide (SIW) based dual band notch UWB antenna.

**Figure 3:**
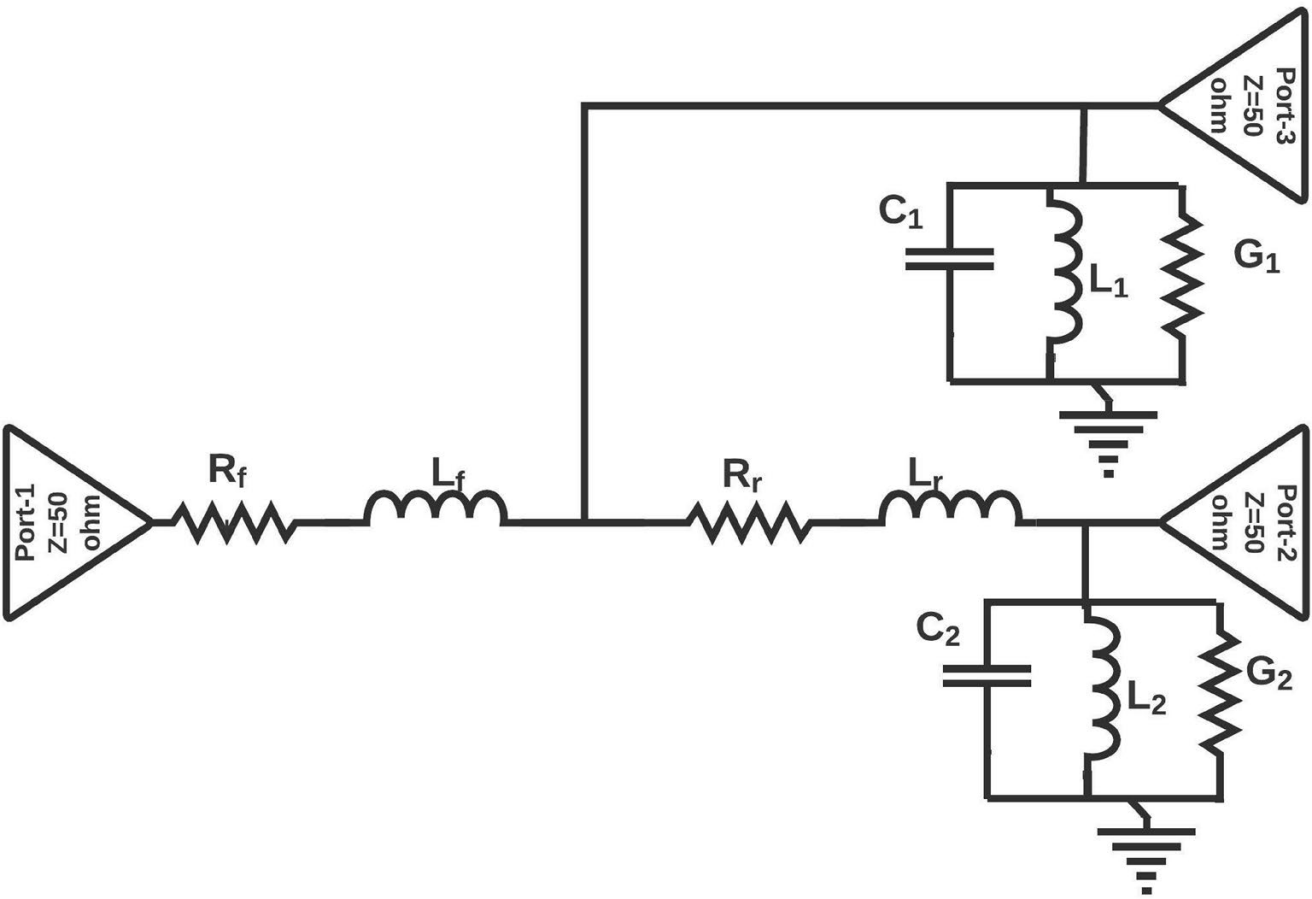
Equivalent circuit model of Narrow band antennas (NB).

**Figure 4:**
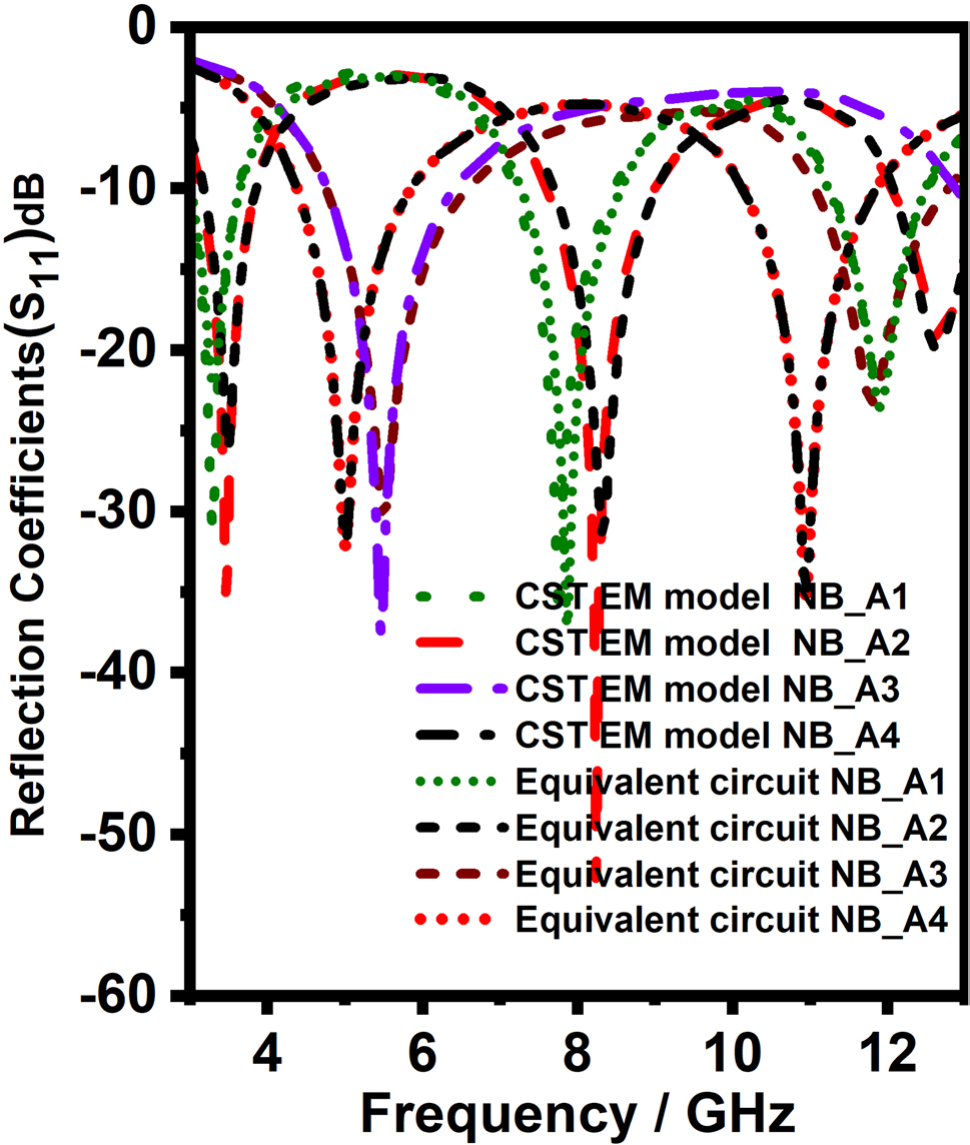
S-parameter comparison between the EM model and the equivalent circuit model for NB antennas.

Figure 2a–d shows the four $\lambda /4$ length NB monopole antennas. It has a feedline, radiating stub, and ground plane, which is responsible for generating the upper, middle, and lower resonance bands. As discussed for generalized microstrip antenna, the equivalent circuit model designed and simulated for the proposed NB antenna. The double and triple bands are resonated due to diversified paths for currents after the feed line. Therefore, as shown in the Fig. 3, these current path’s lumped models are connected in parallel after the equivalent circuit of the feed line. After the optimization process, the values of the lumped elements of the four proposed NBs are obtained. The lumped elements (R, L, C) value of each NB antenna are different because they work at different frequencies. Therefore, at each frequency, the R, L, and C values of the equivalent model should be optimized to match the antenna performance. But, the equivalent circuit models of all NB antennas are identical due to the similar structure. The equivalent circuit model of the proposed NB antenna is derived from the EM model of NB antenna. The equivalent circuit model of NB antenna initiated using a 50$\Omega$ input impedance terminator and R$_{f}$, L$_{f}$ represents 3 mm width feed line, and both are connected in series. Similarly, it can be observed from the Fig. 3 R$_{r}$, L$_{r}$ represents the 3mm width radiating stub, and it is connected in series. Furthermore, G$_{1\ }$and C$_{1}$ represent the equivalent circuit model between feedline and ground plane, and they are connected in parallel. Likewise, G$_{2}$ and C$_{2}$ represent the equivalent circuit model between radiating stub and ground plane, that is connected in parallel. Due to the surface wave and edge field effects, some inductance is created, which is represented by the inductors L$_{1}$ and L$_{2}$. The optimized R, L, C element values presented in Table 1. The final lumped element circuit of the four proposed NB antennas are presented in Fig. 3 ; it can be observed from the Fig. 3 the lumped element circuit of all four NB antennas are identical, but R, L, C, and G value of each NB antennas different as mentioned in Table 1. Consequently, S11 of the equivalent lumped element circuit resembles the EM model S11 at its operating frequency. The S- parameter of the lumped element circuit for four NB antennas shown in the Fig. 4. As shown in the Fig. 4, the lumped element circuit resonates at 3.3, 7.8, 11.8, 3.5, 8.3, 12.3, 5.5, 12.5, 5, and 12 GHz. Also, the Fig. 4 shows the S-parameter comparison between the EM model and the lumped element circuit. The Fig. 4shows a good agreement between the EM model and lumped element circuit S-parameter results since both results have almost the same resonance frequencies. Hence, the proposed lumped element circuit is perfectly matching with the EM model performance.

**Table 1:** Optimized R, L C for the NB antenna’s equivalent circuit model.

Optimized R, L C for the NB antenna (NB_A1) equivalent circuit model					Optimized R, L C for the NB antenna (NB_A3) equivalent circuit model				
R$_{f}$	L$_{f}$	R$_{r}$	L	G$_{1}$	R$_{f}$	L$_{f}$	R$_{r}$	L$_{r}$	G$_{1}$
0.95	0.64	0.6	0.23	0.006	0.95	0.64	0.6	0.23	0.0016
L$_{1}$	C$_{1}$	G$_{2}$	L$_{1}$	C$_{2}$	L$_{1}$	C$_{1}$	G$_{2}$	L$_{2}$	C$_{2}$
0.25	0.36	0.006	0.1	0.36	0.3	0.05	0.0016	0.1	0.05

Optimized R, L C for the NB antenna (NB_A2) equivalent circuit model					Optimized R, L C for the NB antenna (NB_A4) equivalent circuit model				
R$_{f}$	L$_{f}$	R$_{r}$	L$_{r}$	G$_{1}$	R$_{f}$	L$_{f}$	R$_{r}$	L$_{r}$	G$_{1}$
0.95	0.64	0.6	0.23	0.025	0.95	0.64	0.6	0.23	0.0073
L$_{1}$	C$_{1}$	G$_{2}$	L$_{1}$	C$_{2}$	L$_{1}$	C$_{1}$	G$_{2}$	L$_{2}$	C$_{2}$
0.3	0.61	0.025	0.1	0.61	0.3	0.09	0.0073	0.1	0.09

## Equivalent circuit model of UWB antenna

**Figure 5:**
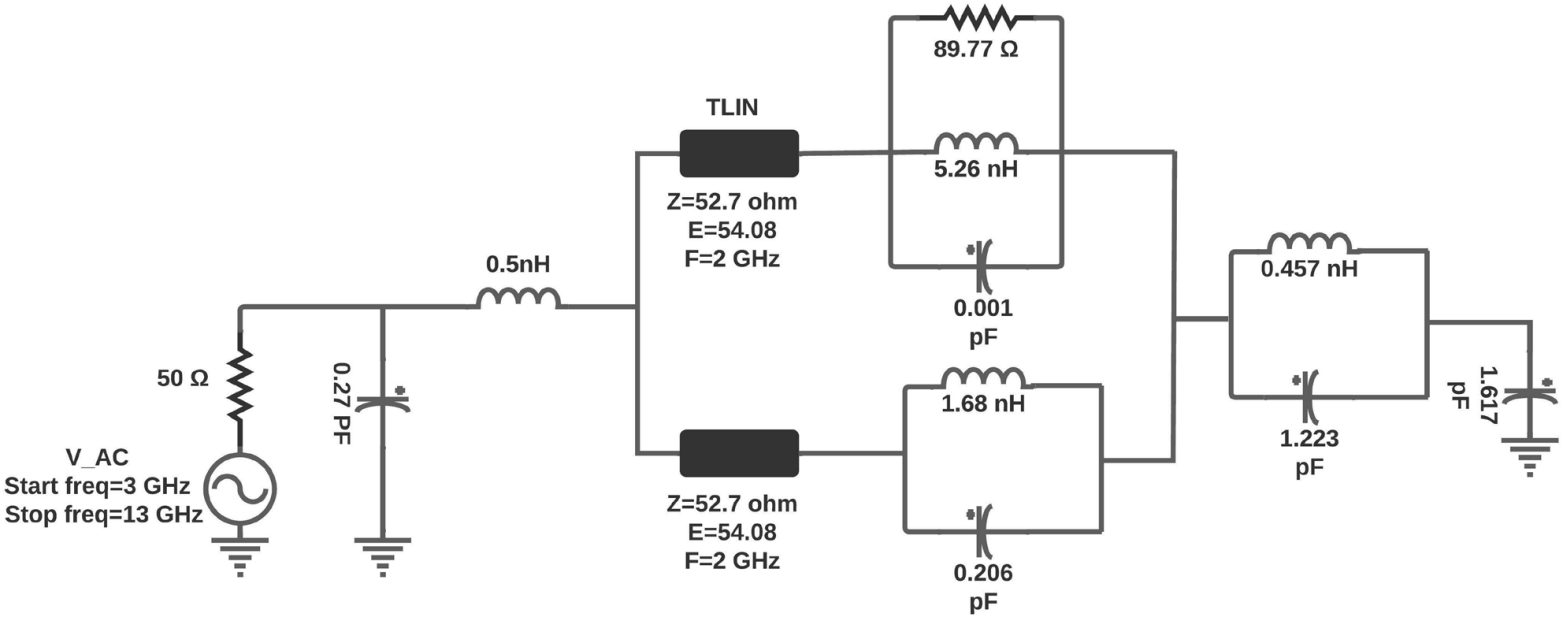
Equivalent circuit model of UWB antenna.

**Figure 6:**
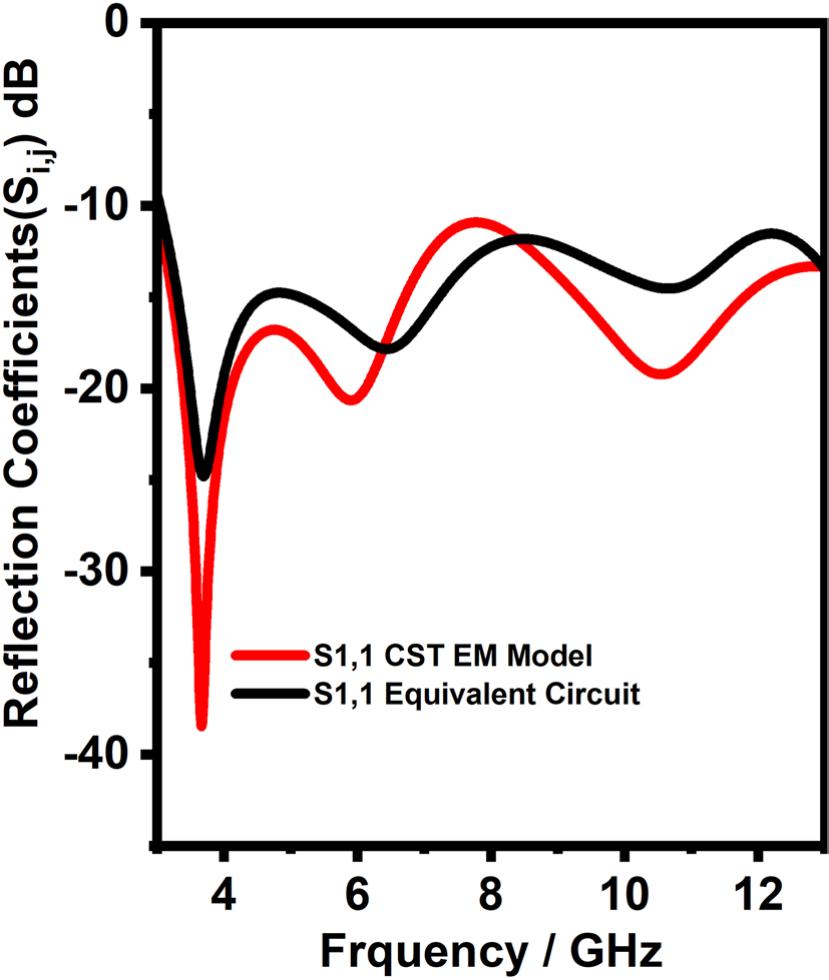
S-Parameter comparison between CST EM model and equivalent circuit model for UWB antenna.

This section describes the lumped equivalent circuit model of UWB antenna. The designed UWB antenna is shown in Fig. 2e. The equivalent circuit model for the UWB antenna is challenging; very few researchers have implemented the equivalent circuit model and found the S parameters. This paper has implemented the equivalent circuit model for the proposed UWB antenna by considering the antenna as a two port network as implemented in^23^. Due to numerous adjacent resonant circuits (represented by parallel RLC circuits), UWB antennas can be modeled according to input impedance characteristics. Also, in this method, we have considered approximately 90-$\Omega$ radiation resistance as a second port, and to represent the distributed elements of the antenna, the transmission lines are incorporated into the equivalent circuit model. In order to match the EM model S-parameter, the equivalent circuit model components values are obtained after so many manual optimization processes. After optimization the final equivalent circuit model for the proposed UWB antenna is shown in the Fig. 5. The proposed UWB antenna equivalent circuit model was optimized and simulated in the Advanced Design System (ADS) software. The transmission line is included in the equivalent circuit to represent the distributed elements of the antenna. Figure 6 Shows the comparison of S11 between the equivalent circuit model and the CST EM model. It can be seen from Fig. 6 that the two S-parameters for both cases having good agreement.

## Equivalent circuit of half wave length band notch resonator

The designed UWB antenna with band notch filter is shown in Fig. 2f. In order to study the principles behind the equivalent circuit model of the half-wavelength notch-band resonator, it is derived from the antenna input impedance, which is obtained from the CST EM solver. Further, in order to achieve band notch characteristics, the antenna should be high impedance mismatch at the notch band. From the simulation or measurement result corresponding to resonance (Z$_{La}$), and the continuous parameter values (R$_{j}$, L$_{j}$, C$_{j}$) the equivalent circuit model are computed. The $\omega$ $_{SR}$, $\omega$ $_{PR}$, BW$_{SR}$, and BW$_{PR\ }$are series resonance, parallel resonance, series resonance bandwidth, and parallel resonance bandwidth, respectively, which are obtained from the input impedance (Z$_{la}$) by using Eqs. (1)–(5). Besides, R$_{sn}$ and R$_{pn}$ are acquired from input impedance.

1 $$\begin{aligned} Z_{la}={\textstyle \sum _{J=1}^{n}}\frac{j\omega R_jL_j}{R_j \left( 1-\omega ^{2}L_jC_j\right) +j\omega L_j} \end{aligned}$$

The equivalent circuit component values L$_{sn}$, C$_{sn}$, L$_{pn}$, and C$_{pn}$ are computed using the equation with recorded values, where n = 1, 2, 3...

2 $$\begin{aligned}&\omega _{SR}=\frac{1}{\sqrt{{\mathrm L}_{sn}\times {\mathrm C}_{sn}}} \end{aligned}$$

3 $$\begin{aligned}&BW_\mathrm {SR}=\frac{{\mathrm R}_\mathrm {sn}}{{\mathrm L}_\mathrm {sn}} \end{aligned}$$

4 $$\begin{aligned}&\omega _\mathrm {PR}=\frac{1}{\sqrt{{\mathrm L}_\mathrm {pn}\times {\mathrm C}_\mathrm {pn}}} \end{aligned}$$

5 $$\begin{aligned}&BW_\mathrm {PR}=\frac{1}{\mathrm R}_\mathrm {pn}\times {\mathrm C}_\mathrm {pn} \end{aligned}$$

### Equivalent circuit of L-shaped parasitic stub notch resonators (WLAN notch)

**Figure 7:**
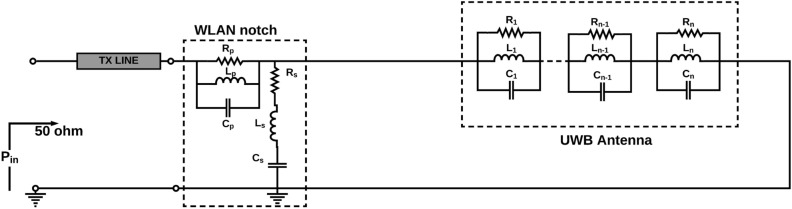
The lumped equivalent circuit model of WLAN(5 GHz) band notch filter.

In the case of the WLAN frequency band, at 5 GHz and 5.25 GHz, the actual impedance is 200 $\Omega$ and 23 $\Omega$, respectively. Also, the imaginary value of the impedance fluctuates from +ve to -ve value for both frequencies. The equivalent circuit for the WLAN band notch resonator is obtained by combining impedance analysis at both frequencies, and it consists of a series and parallel combination RLC components. The lumped equivalent circuit model of WLAN (5 GHz) band notch filter is shown Fig. 7. The optimized lumped elements (R, L, C) values shown in Table 2. It can be seen from the EM model and the lumped circuit model S-parameters are almost the same. Also, it is witnessed that at the notch band the S11 is greater than -2dB for both EM model and equivalent circuit model due to impedance mismatch. Equation (6) used to achieve band notch at WLAN band. Hence, it is clear from the reflection coefficient the WLAN band notch is effectively achieved by using an L-shaped stub.

6 $$\begin{aligned}&F_{WLANnotch}=\frac{c}{4\sqrt{\varepsilon _{eff}}\left( L_{WLAN}\right) } \end{aligned}$$

### Equivalent circuit of folded L-shaped stub notch resonators (ITU-band notch)

**Figure 8:**
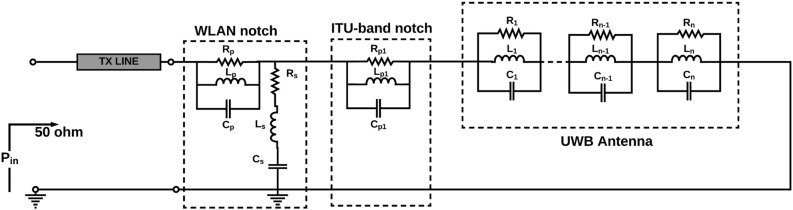
The lumped equivalent circuit model of WLAN and ITU (8 GHz) band notch filter.

**Figure 9:**
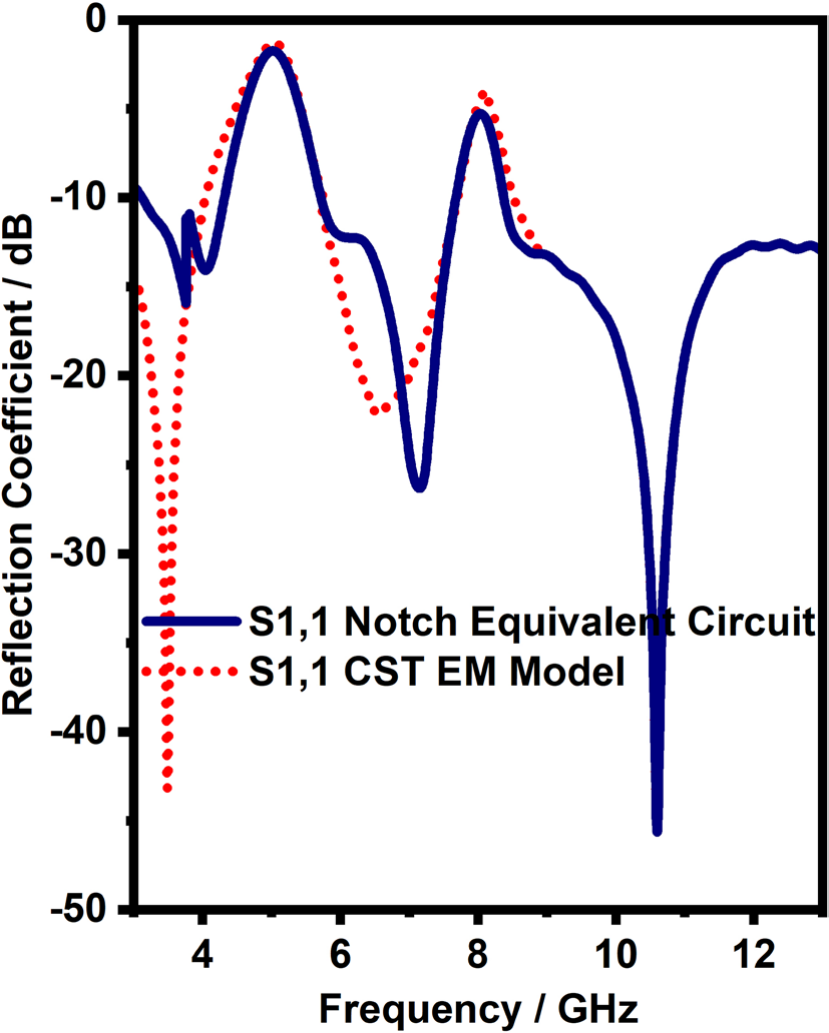
EM model and the lumped circuit model S-parameters for dual band notch (5 and 8 GHz) filter.

For the ITU frequency band, at 8 GHz, the real impedance is 200 $\Omega$. Also, the imaginary value of the impedance varies from +ve to −ve value at 8 GHz. The equivalent circuit for the ITU band notch resonator consists of a series and parallel combination RLC components. The lumped equivalent circuit model of ITU (8 GHz) band notch filter is shown Fig. 8. The optimized lumped elements (R, L, C) values shown in Table 2. It can be seen from Fig. 9 the EM model and the lumped circuit model S-parameters are almost the same. Also, it is witnessed that at the notch band the S11 is greater than − 2 dB for both EM model and equivalent circuit model due to impedance mismatch. Equation (7) used to achieve band notch at WLAN band. Hence, it is clear from the reflection coefficient the ITU band notch is effectively achieved by using a two L-shaped slot.

7 $$\begin{aligned}&F_{{Xband notch}}=\frac{c}{4\sqrt{\varepsilon _{eff}}\left( L_{{X-band}}\right) } \end{aligned}$$

**Table 2:** Optimized RLC components for notched bands (R in $\Omega$, L in nH, and C in PF).

R$_{p\ }$	L$_{p\ }$	C$_{p\ }$	R$_{s\ _{}}$	L$_{s}$	C$_{s}$	R$_{s1}$	L$_{s1}$	C$_{s1}$
286	0.065	4.67	31.2	28.96	0.052	312	0.693	2.69

**Figure 10:**
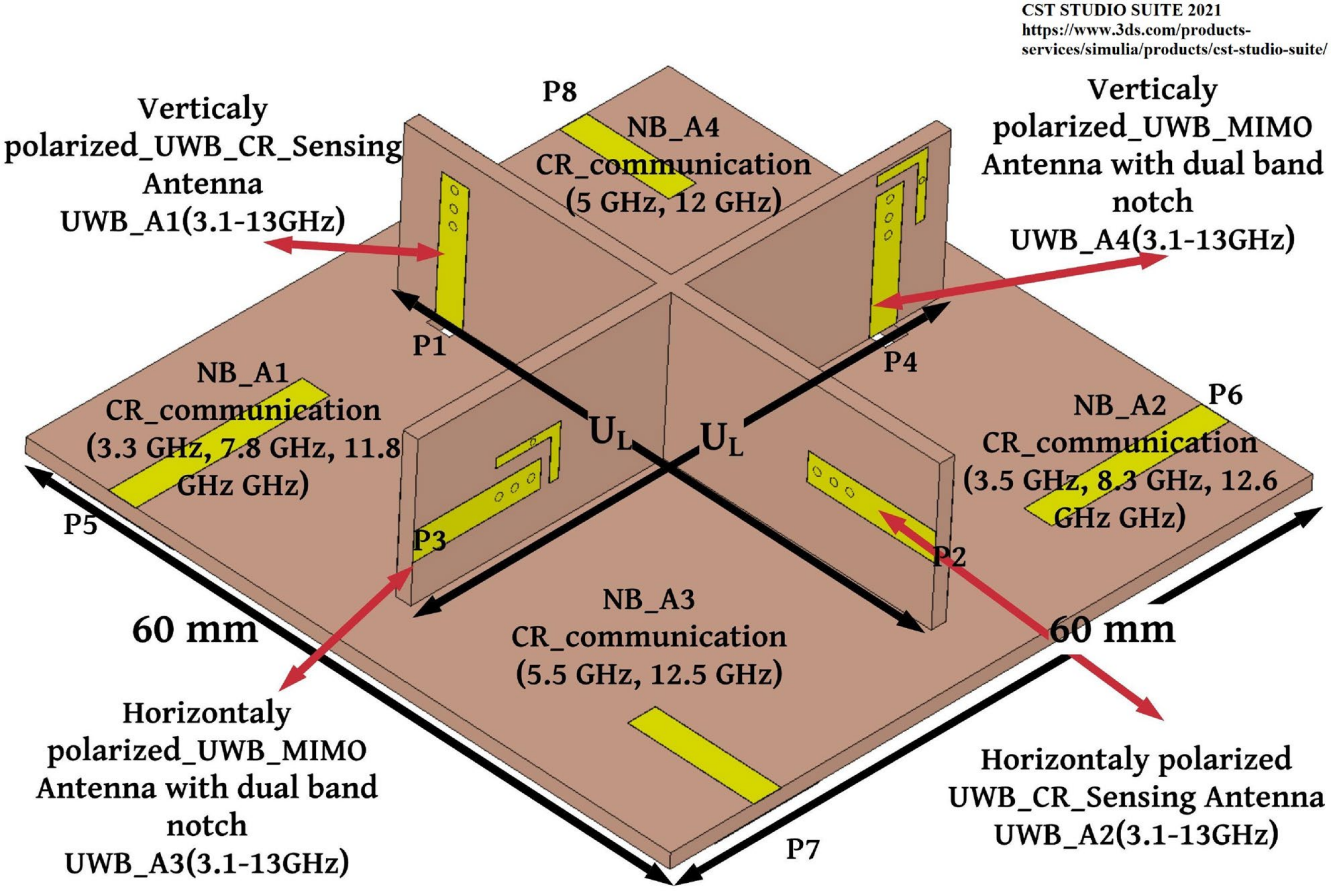
Integrated antenna system. (CST STUDIO SUITE 2021).

## Working of the integrated antenna system

The hybrid antenna system includes vertically and horizontally polarized UWB sensing radiator, UWB MIMO antenna, and horizontal polarized NB communication antennas for CR and UWB MIMO communication as depicted in Fig. 10. The Metallic Via(M-Via)-based UWB radiators are connected in ports 1 and 2 to identify the idle spectrum in the 3–13 GHz and communications antennas are connected in ports 5, 6, 7, and 8 which operates at ten resonant frequencies (3.3, 7.8, 11.8, 3.5, 8.3, 12.6, 5.5, 12.8, 5 and 12 GHz) as depicted in Fig. 10. Further vertically and horizontally polarized M-Via-based UWB radiator with dual-band notch is used for $2 \times 2$ UWB MIMO antenna and it is attached with ports 3 and 4 as depicted in Fig. 10.

### Operational configurations of the integrated antenna

Based on the idle spectrum identification in the 3–13 GHz band, the integrated antenna system can work in six different configurations. For all six operating conditions, the sensing radiators and UWB MIMO radiators are being active to identify unoccupied frequencies in the 3–13 GHz band and MIMO applications respectively. But the four NB antennas are always being in idle and it is activated based on the white space detection.

#### Operational configuration 1

If the UWB sensing radiators identify white space at 3.3, 7.8, and 11.8 GHz the NB-A1 will be activated for CR communication purposes during this time other NB antennas are being inactive.

#### Operational configuration 2

If the UWB sensing radiators identify white space at 3.5, 8.3, and 12.3 GHz the NB-A2 will be activated for CR communication purposes during this time other NB antennas are being inactive.

#### Operational configuration 3

If the UWB sensing radiators identify white space at 5.5, and 12.8 GHz the NB-A3 will be activated for CR communication purposes during this time other NB antennas are being inactive.

#### Operational configuration 4

If the UWB sensing radiators identify white space at 5, and 12 GHz the NB-A4 will be activated for CR communication purposes during this time other NB antennas are being inactive.

## Simulation and measurement results analysis of the integrated antenna system

**Figure 11:**
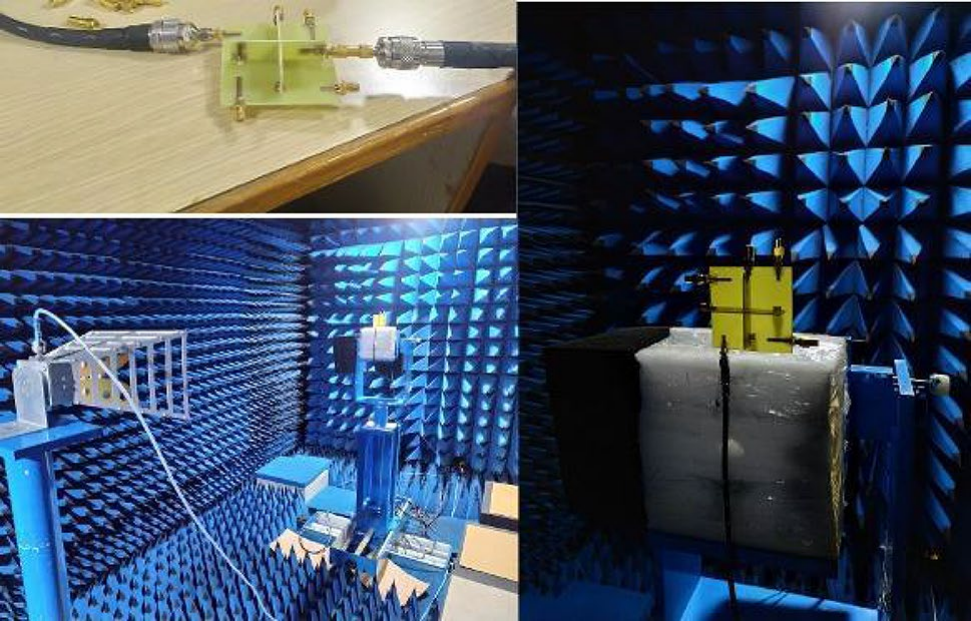
Photo of fabricated antenna and measurement setup.

**Figure 12:**
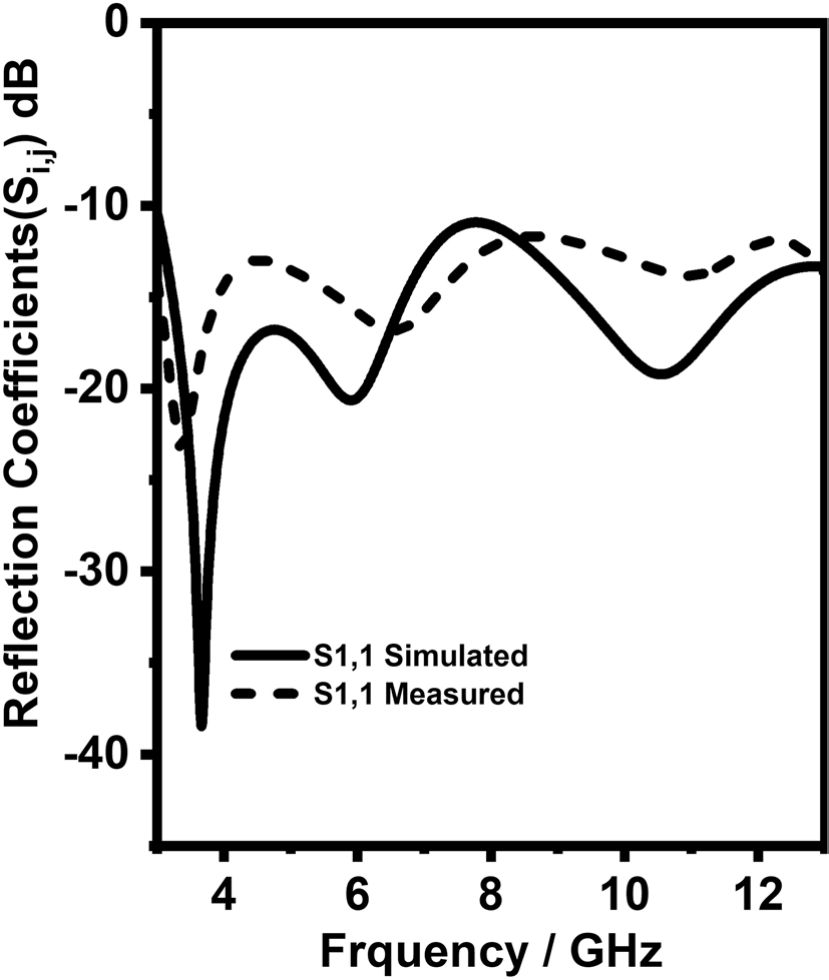
S-Parameters of UWB radiator (Simulated and measured).

**Figure 13:**
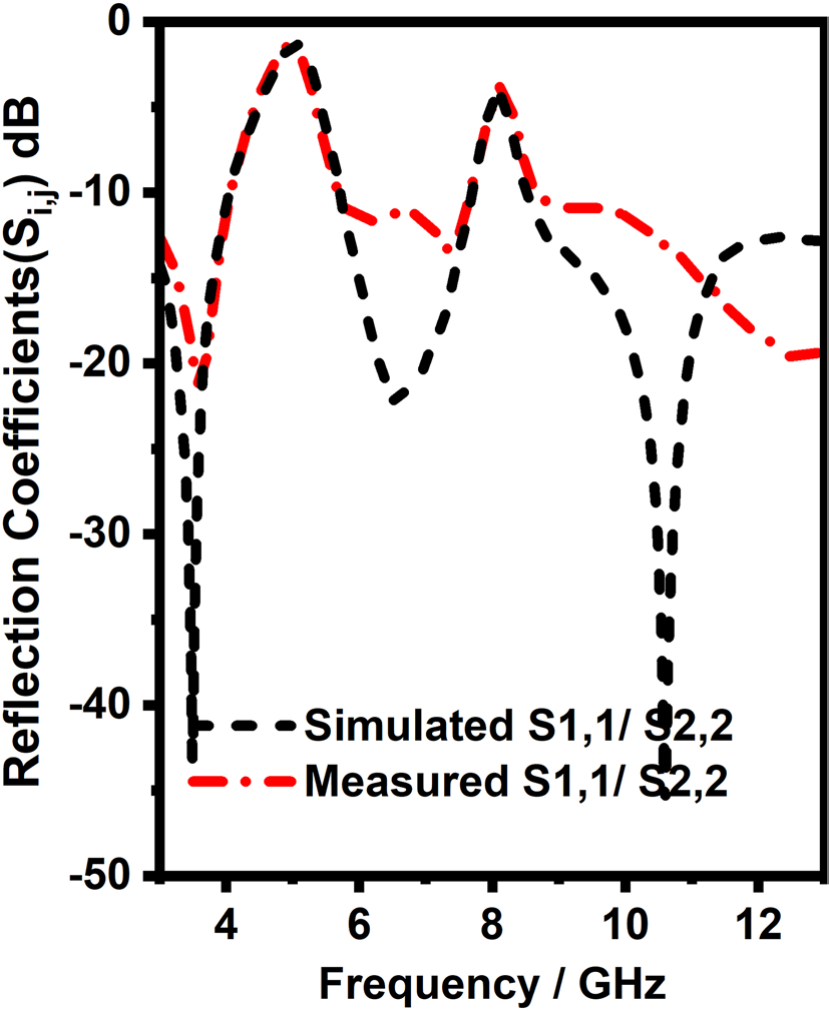
Reflection coefficients (Simulated and measured) of 2$\times$2 element UWB MIMO antenna.

**Figure 14:**
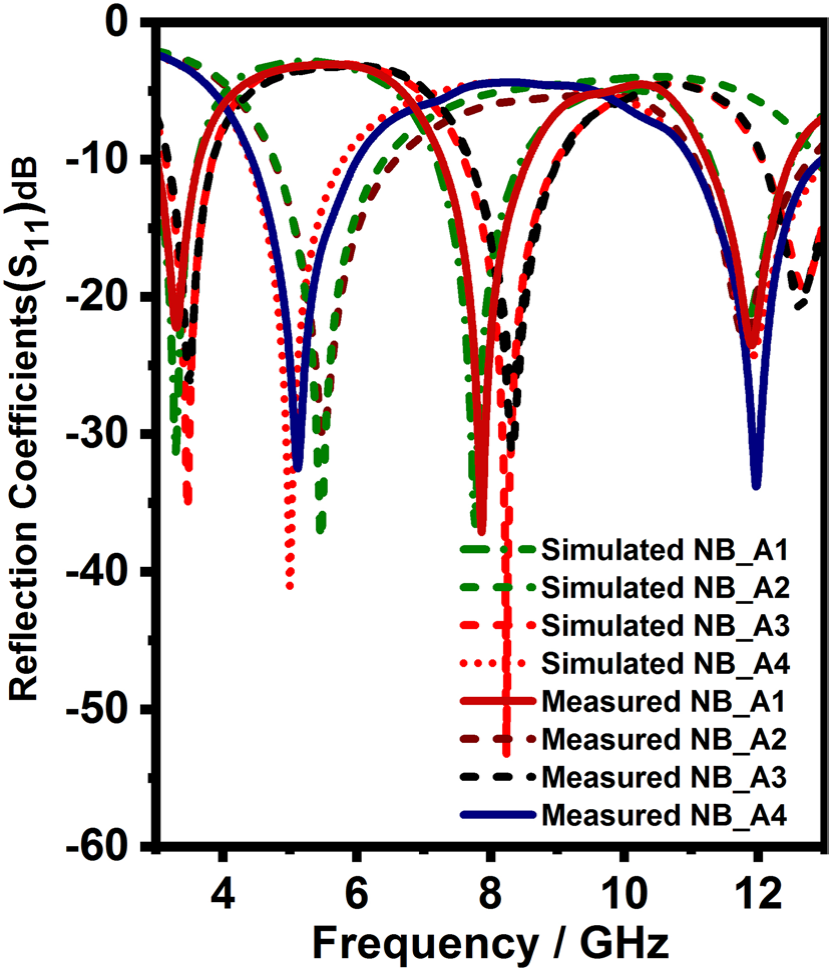
Reflection coefficients (Simulated and measured) NB antenna.

After equivalent circuit analysis and EM model analysis, the proposed antenna is fabricated and tested in Vector Network Analyzer(VNA) and anechoic chamber to verify the performance of the antennas as depicted in Fig. 11. Figures 12, 13 and 14 compares the simulated and measured antenna parameters of the integrated antenna system.

Initially, Fig. 12 shows the simulated and measured reflection coefficients of the vertically and horizontally polarized UWB sensing radiators and it can be witnessed from Fig. 12 it has 3–13 GHz bandwidth with less than − 10 dB. Secondly, Fig. 14 shows the simulated and measured reflection coefficients of the NB communication antenna and it can be witnessed from Fig. 14 it has ten resonant frequencies (3.3, 7.8, 11.8, 3.5, 8.3, 12.3, 5.5, 12.5, 5, and 12 GHz) over 3–13 GHz with less than − 10 dB. Thirdly, Fig. 13 shows the simulated and measured reflection coefficients of the vertically and horizontally polarized two elements UWB MIMO antenna and it can be witnessed from Fig. 13 it has 3–13 GHz bandwidth with less than − 10 dB except for notch bands(5 and 8 GHz).

**Figure 15:**
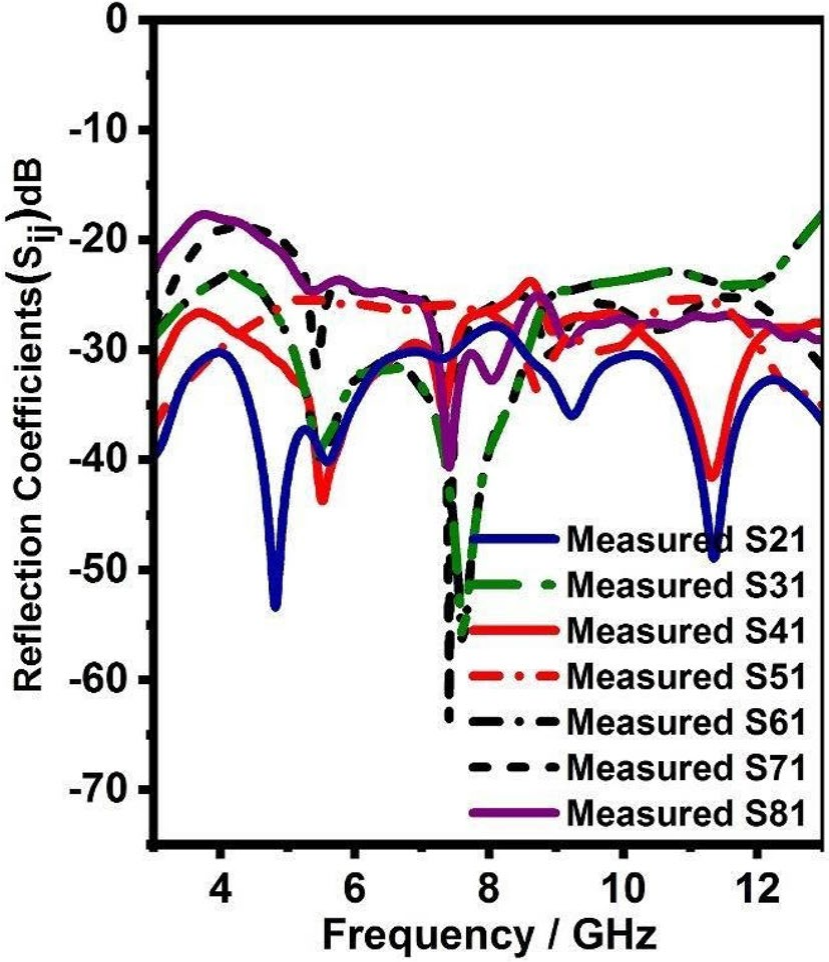
Mutual coupling (Measured) of integrated antenna system.

**Figure 16:**
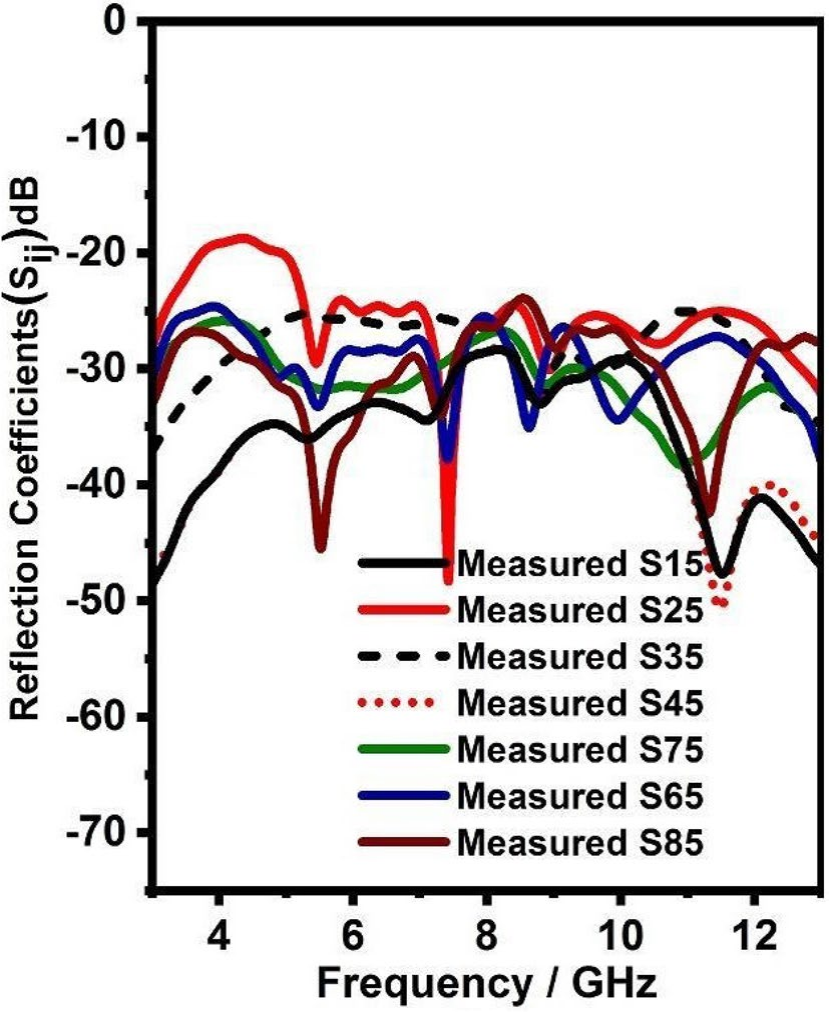
Mutual coupling (Measured) of integrated antenna system.

Further, Figs. 15 and 16 shows the simulated and measured mutual coupling of the eight-port integrated antenna and it can be witnessed from Figs. 15 and 16 it has less than − 20 dB mutual coupling over 3–13 GHz.

**Figure 17:**
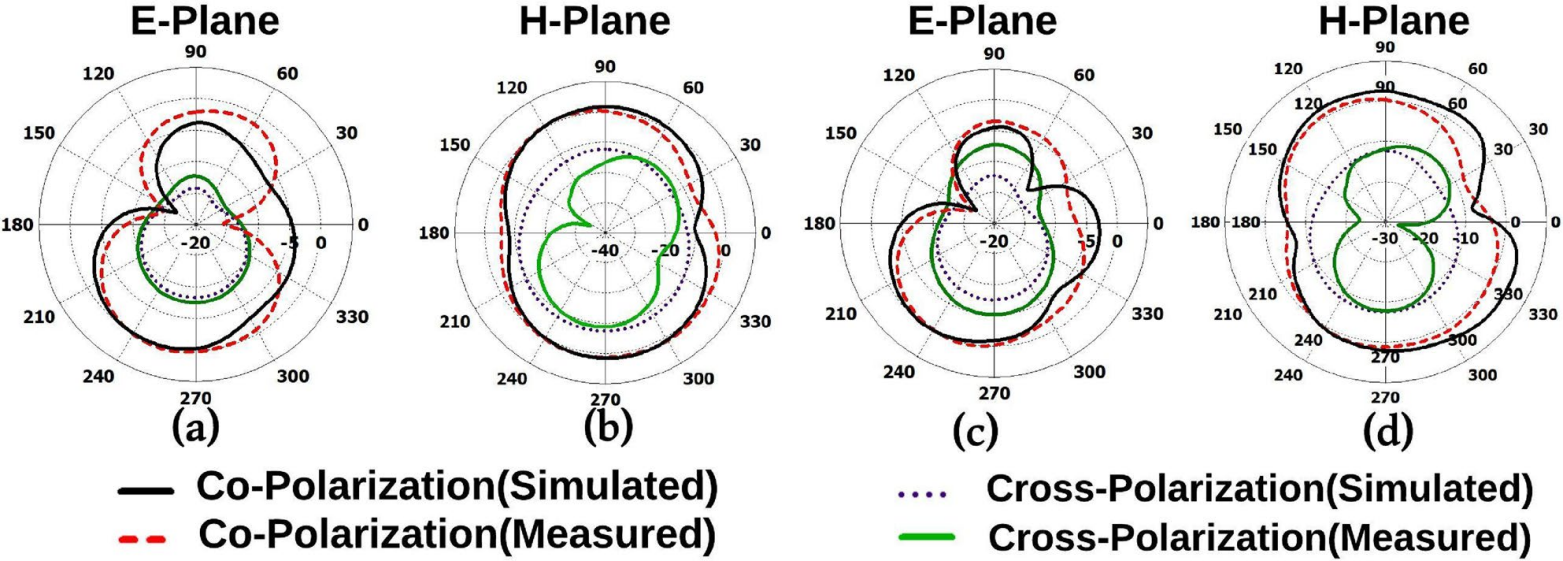
Radiation pattern (simulated and measured) for UWB radiators.

**Figure 18:**
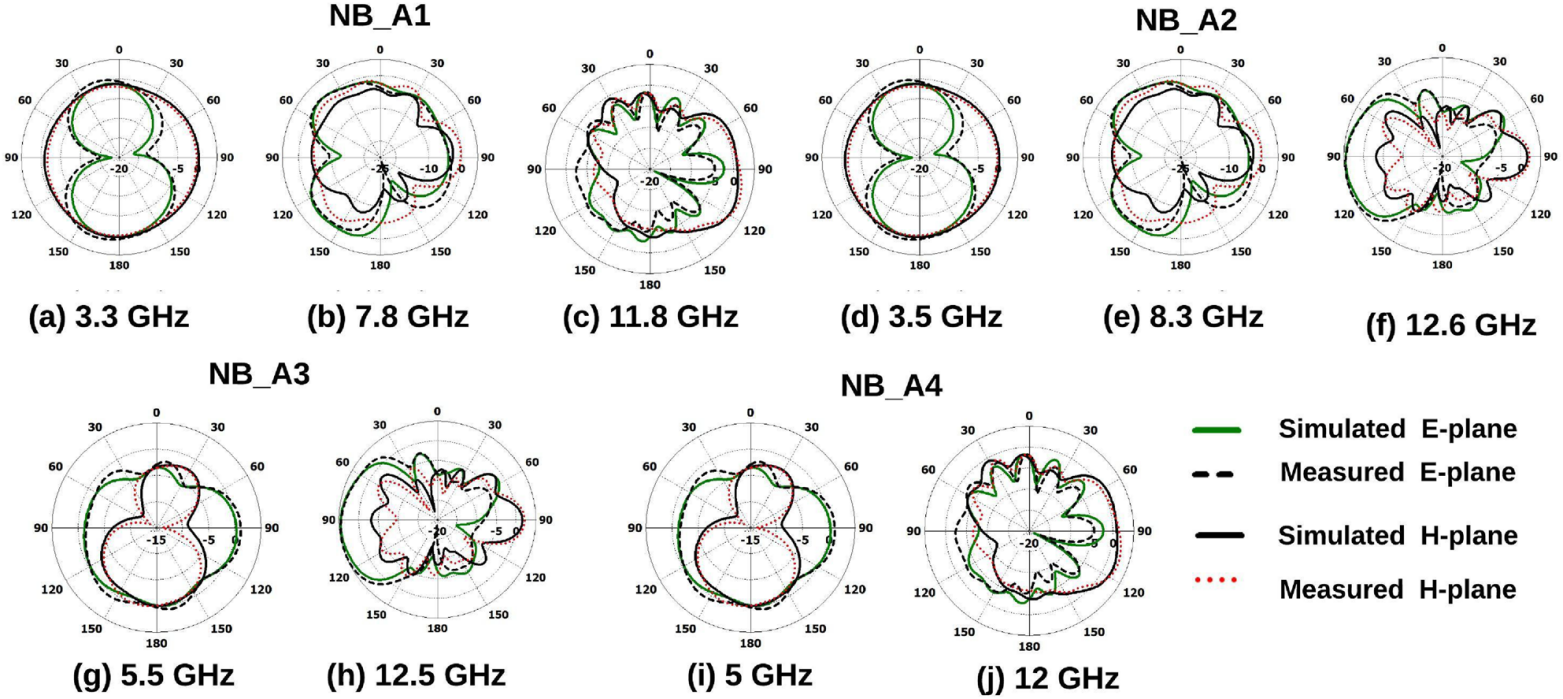
Radiation pattern of NB antennas.

The radiation pattern(simulated and measured) of the UWB sensing radiator, UWB MIMO radiator, and NB radiators are illustrated in Figs. 17a–d and 18a–j. It can be witnessed from the Fig. 17a–d the UWB sensing antenna and UWB MIMO antenna radiator have omnidirectional radiation at 3.5, 6.5, 9, and 10 GHz . Similarly, as witnessed from the Fig. 18a–j the NB antenna has an almost omnidirectional radiation pattern at all NB resonant frequencies (3.3, 7.8, 11.8, 3.5, 8.3, 12.6, 5.5, 12.5, 5, and 12 GHz).

**Figure 19:**
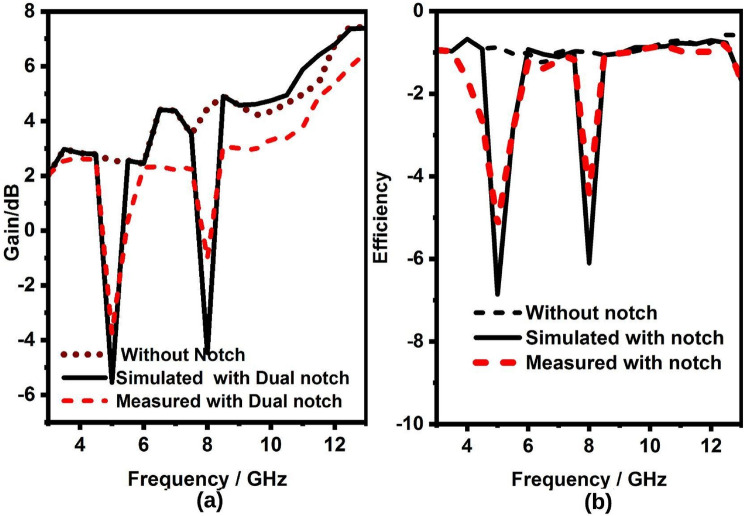
UWB sensing radiator (**a**) gain, (**b**) efficiency, 2$\times$2 band notch UWB MIMO antenna.

**Figure 20:**
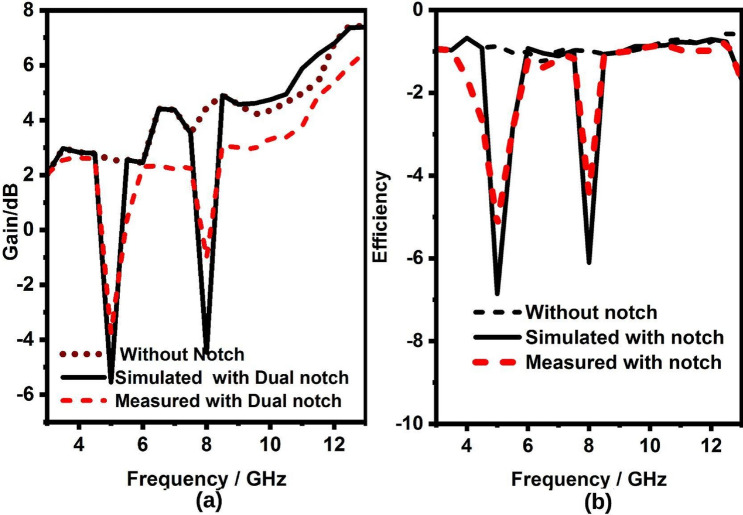
2$\times$2 band notch UWB MIMO antenna (**a**) gain, (**b**) efficiency.

Further, the gain is varying from 2–75 to 3–7.6 dB but for the notch band, it is less than − 3.4 dB, for sensing radiator and two elements MIMO antenna radiator respectively as shown in Fig. 19a,b. Likewise, the efficiency is varying from 70–80%, and 71–85% except for the notch band but at the notch band, it is less than 45%, for sensing radiator and two elements MIMO antenna radiator respectively as shown in Fig. 20a,b. Finally, there is a clear correlation between the simulated and measured antenna parameters. However, minor variations are observed due to manufacturing and soldering errors, but they are tolerable.

## MIMO parameter analysis of $2\times 2$ UWB MIMO antenna

As part of an integrated antenna system, the $2\times 2$ UWB MIMO antenna is used for UWB MIMO operations. A $2\times 2$ UWB MIMO antenna’s MIMO performance is evaluated using MIMO parameters. MIMO parameters are given as a table instead of a plot for the purpose of simplicity and to make it easier to read. As indicated in Table 3, four frequencies (3.5, 6.5, 9, and 10 GHz) are selected for study in the 3–13 GHz range.

**Table 3:** MIMO diversity analysis of 2-element UWB MIMO antenna: (Mutual Coupling (MC), Envelope Correlation Coefficient (ECC), Total Active Reflection Coefficient (TARC), and Channel Capacity Loss (CCL) are measured).

f GHz	ECC	MC dB	DG	TARC			CCL
3.5	0.004	$<-22$	9.97	$\theta$= 0= <− 20	$\theta$= 30= <− 20	$\theta$= 60= <− 20	< 0.4
6.5	0.004	<− 28	10	$\theta$= 0= <− 20	$\theta$= 30= <− 20	$\theta$= 60= <− 20	< 0.4
9	0.003	<− 28	9.97	$\theta$= 0= <− 20	$\theta$= 30= <− 20	$\theta$= 60= <− 20	< 0.4
10	0.002	<− 23	10	$\theta$= 0= <− 20	$\theta$= 30= <− 20	$\theta$= 60= <− 20	< 0.4

From Table 3 the proposed polarization diversity antenna has a DG value of greater than 9.9 dB, ECC of 0.005, TARC value of less than − 20 dB, CCL of less than 0.5 bit/Hz/s across the operating bandwidth (3–13 GHz). As a result, the vertical and horizontal orientations of the radiators in the proposed integrated antenna system offer better diversity performance.

## Functional verification of CR and UWB MIMO antenna

**Figure 21:**
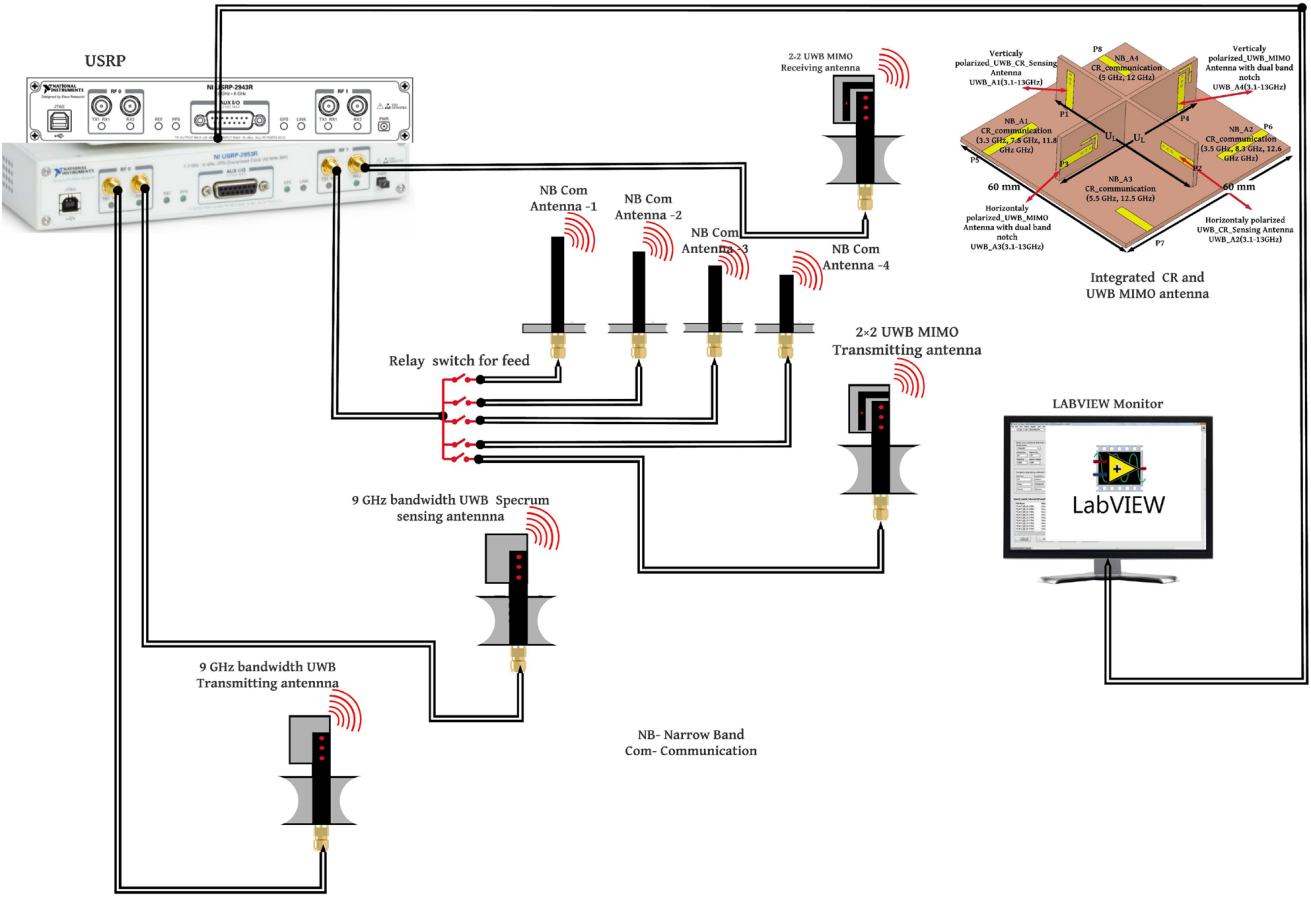
Block diagram of the proposed Hardware implementation.

This section describes the hardware connection, wide band spectrum generation, and detection. The spectral sensing and communication capabilities of the designed integrated CR antenna is evaluated using a fabricated prototype of the antenna, NI USRP 2943R, the bayesian learning algorithm and NI LABVIEW software monitor. Figure 21 depicts a block diagram of the USRP-based 3 GHz (3–6 GHz) spectrum generation, spectrum detection block, white space detection, and communication systems. USRP device with proposed antenna is used for spectrum generation, detection, and communication function verification. The USRP has two channels: RF0 and RF1, and each channel (RF0 and RF1) has two ports. Furthermore, the device can transmit and receive spectrum ranging from 1.2 to 6 GHz. The proposed CR and UWB MIMO functional verification block diagram is shown in Fig. 21.

In order to verify the CR antenna system functional verification following procedure is followed

- A UWB antenna is connected to an RF0 port to generate a 3 GHz (3–6 GHz) wide spectrum.
- Another UWB antenna is linked to the USRP RF0 Rxr port to detect a 3 GHz (3–6 GHz) spectrum, and four NB communication antennas are linked to USRP RF1.
- The NB communication(NB Com) antennas operate at 3.3 (NB_A1) GHz, 3.5 GHz (NB_A2), 5 GHz (NB_A3) and 5.5 GHz (NB_A4).

**Figure 22:**
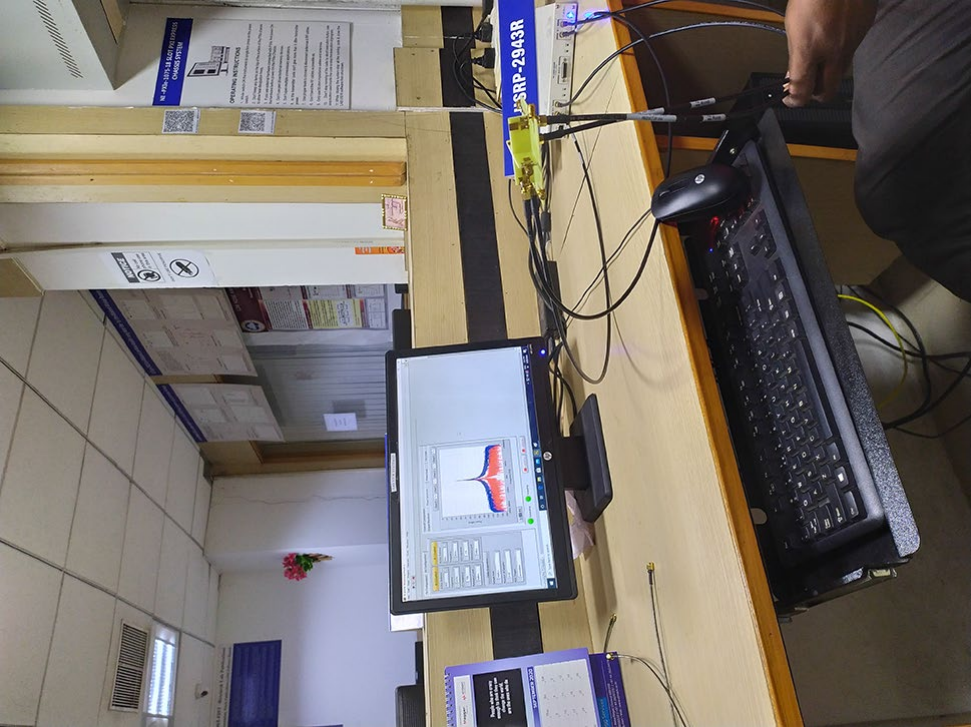
Real time implementation lab set-up.

**Figure 23:**
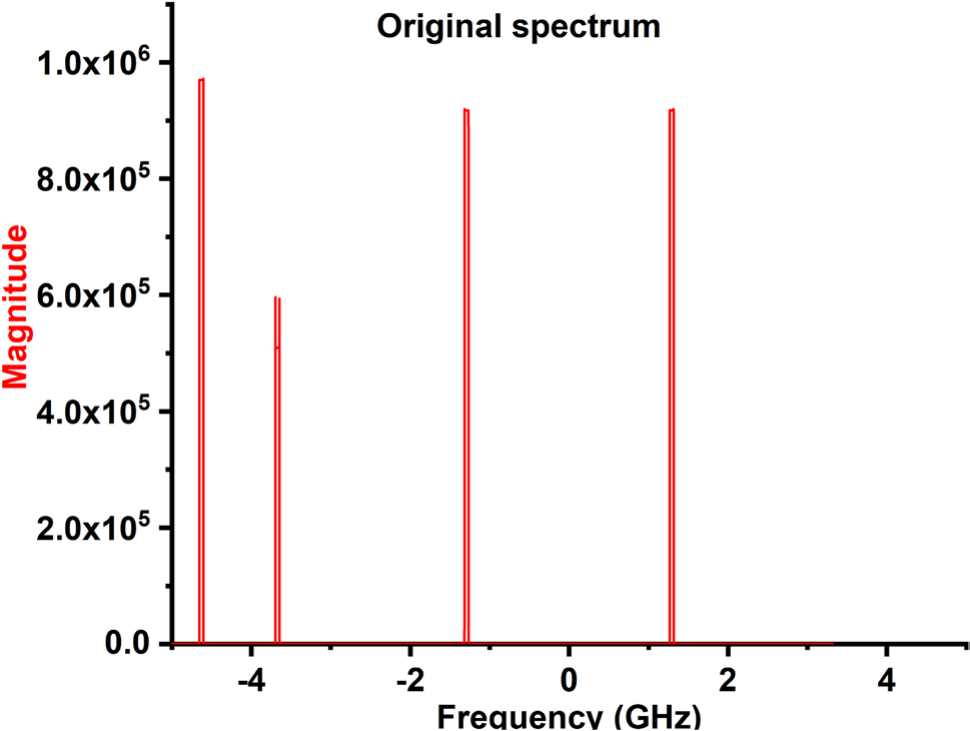
Detected 3.8 GHz spectrum after processed in MATLAB. (Matlab V2021).

**Figure 24:**
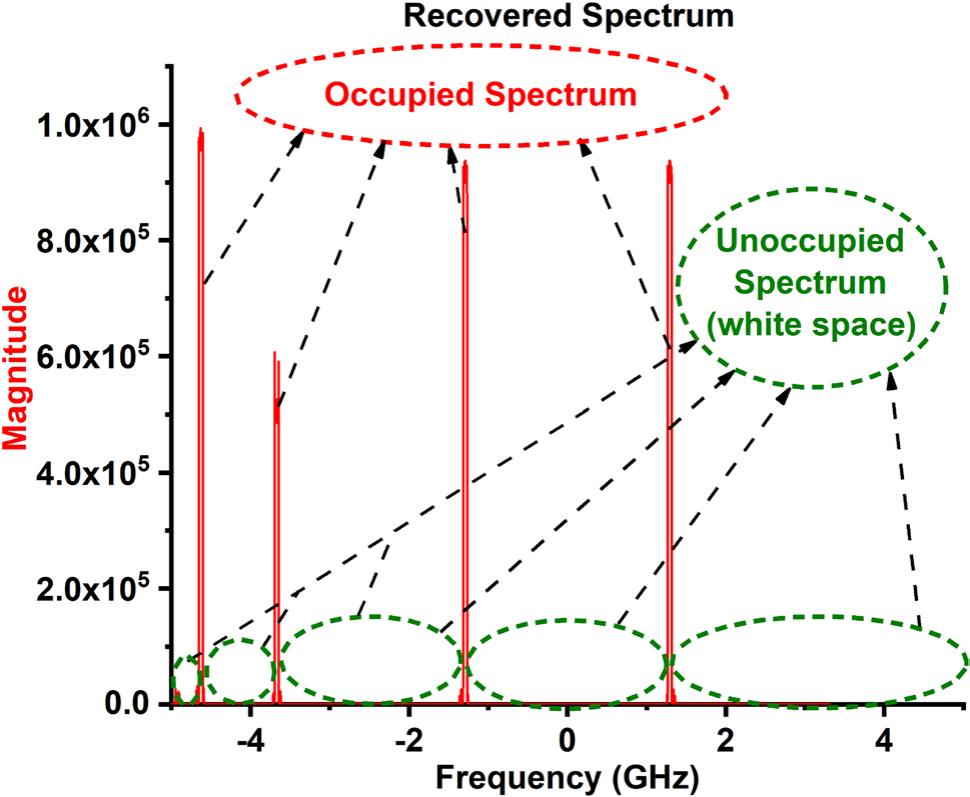
Recovered spectrum after processed using Bayesian learning-based spectrum sensing algorithm in MATLAB. (Matlab V2021).

**Figure 25:**
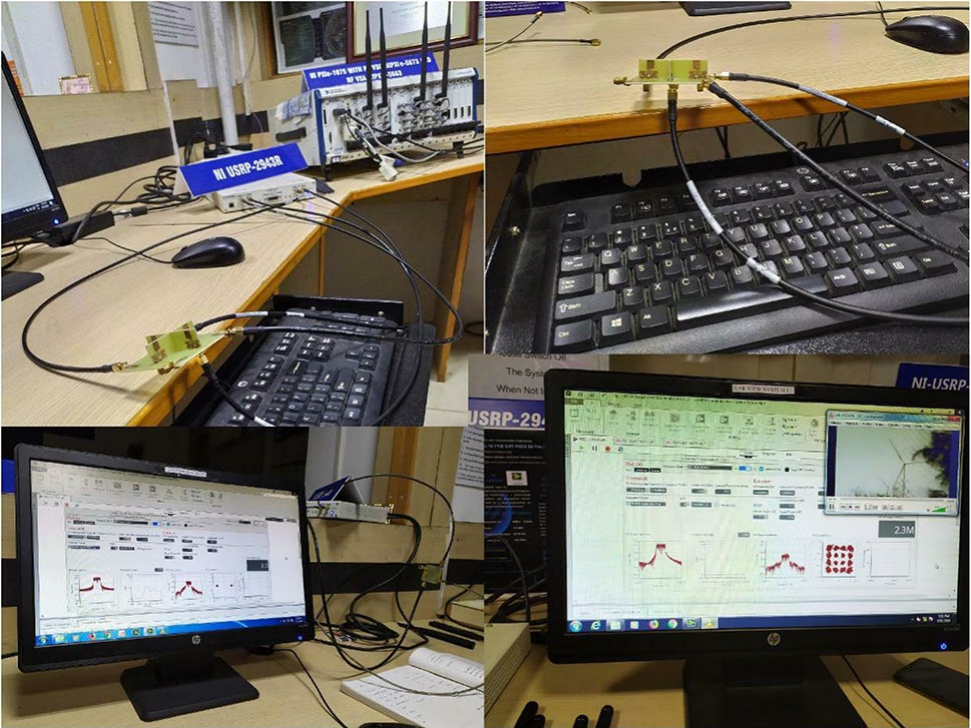
Functional verification lab set up of the two element UWB MIMO antenna.

- The relay switch is used to activate the corresponding NB communication antennas according to the white space detection(In this work switching is done manually).
- The transmitting UWB antenna generates a 3 GHz (3–6 GHz) bandwidth spectrum by exciting the UWB antenna through the USRP RF0 Tx port.
- Then, the receiving UWB antenna detects the generated 3 GHz (3–6 GHz) wide spectrum and feeds it to the USRP RF0 Rx port.
- A real-time algorithm is used to visualize the USRP received signal in LABVIEW and it is shown in Fig. 22.
- In addition, GNU Radio and MATLAB software are utilized to execute processing on the PC using an FFT-based algorithm to generate the 3 GHz (3–6 GHz) spectrum as shown in Fig. 23 .
- The processed 3 GHz (3–6 GHz) spectrum is shown in the Fig. 23. Further, in order to find the white space in the detected 3 GHz (3–6 GHz) spectrum, we have used Bayesian learning-based spectrum sensing algorithm in MATLAB.

- After the process the occupied and free spectrum is identified as depicted in Fig. 24. It is witnessed from the figure there are five white space in this detected spectrum, which is at 3.3, 3.5, 5, and 5.5 GHz.
- Finally, the Narrow band antennas are activated manually as depicted in Figs. 21 and 22 and the communication is carried out at detected white space (3.3, 3.5, 5, and 5.5 GHz).

- As shown in Fig. 21, in order to verify the real time short range video transmission and reception capability of the two element UWB MIMO antenna, the two UWB MIMO antenna is connected to RF1 channel of USRP’s. First UWB MIMO antenna is considered as transmitter and second UWB MIMO antenna is considered as receiver and lab set up of the video transmission and reception is shown in Fig. 25.

- After the connection video signal is transmitted through first UWB MIMO antenna and received by the second UWB MIMO antenna. The transmitted and received video signal is visualized in LABVIEW monitor as depicted in Fig. 25. It can be observed that it has throughput of 106 Mb/s.

## Conclusion

In this article, the equivalent circuit models have been proposed for NB antennas, UWB antenna, and band notch filters. The lumped element model for the UWB antenna is achieved after many optimizations. The lumped element’s value is calculated approximately and optimized in AWR, ADS, and CST to obtain an accurate lumped element (R, L, and C) value. These equivalent circuit models of NB antennas, UWB antenna, and band notch filters show identical performance between the equivalent circuit model and EM model results. The simulated and measured results are analyzed using important antenna parameters. Also, the functional verification of the integrated antenna is carried out using USRP, and it is witnessed that the integrated antenna is suitable for CR spectrum sensing and communication applications and short range video transmission applications.

## Data Availability

The datasets used and/or analysed during the current study available from the corresponding author on reasonable request.
